# The Utility of Liver Function Tests for Mortality Prediction within One Year in Primary Care Using the Algorithm for Liver Function Investigations (ALFI)

**DOI:** 10.1371/journal.pone.0050965

**Published:** 2012-12-14

**Authors:** David J. McLernon, John F. Dillon, Frank M. Sullivan, Paul Roderick, William M. Rosenberg, Stephen D. Ryder, Peter T. Donnan

**Affiliations:** 1 Division of Applied Health Sciences, University of Aberdeen, Aberdeen, United Kingdom; 2 Biomedical Research Institute, Ninewells Hospital and Medical School, University of Dundee, Dundee, United Kingdom; 3 Division of Population Health Sciences, Medical Research Institute, University of Dundee, Dundee, United Kingdom; 4 Academic Unit of Primary Care and Population Sciences, Faculty of Medicine, University of Southampton, Southampton, United Kingdom; 5 Centre for Hepatology, Division of Medicine and ULCH-UCL NIHR Biomedical Research Centre, University College London, London, United Kingdom; 6 Department of Gastroenterology, Nottingham University Hospitals NHS Trust and Biomedical Research Unit, Nottingham, United Kingdom; University of Colorado, United States of America

## Abstract

**Background:**

Although liver function tests (LFTs) are routinely measured in primary care, raised levels in patients with no obvious liver disease may trigger a range of subsequent expensive and unnecessary management plans. The aim of this study was to develop and validate a prediction model to guide decision-making by general practitioners, which estimates risk of one year all-cause mortality in patients with no obvious liver disease.

**Methods:**

In this population-based historical cohort study, biochemistry data from patients in Tayside, Scotland, with LFTs performed in primary care were record-linked to secondary care and prescription databases to ascertain baseline characteristics, and to mortality data. Using this derivation cohort a survival model was developed to predict mortality. The model was assessed for calibration, discrimination (using the C-statistic) and performance, and validated using a separate cohort of Scottish primary care practices.

**Results:**

From the derivation cohort (n = 95 977), 2.7% died within one year. Predictors of mortality included: age; male gender; social deprivation; history of cancer, renal disease, stroke, ischaemic heart disease or respiratory disease; statin use; and LFTs (albumin, transaminase, alkaline phosphatase, bilirubin, and gamma-glutamyltransferase). The C-statistic for the final model was 0.82 (95% CI 0.80–0.84), and was similar in the validation cohort (n = 11 653) 0.86 (0.79–0.90). As an example of performance, for a 10% predicted probability cut-off, sensitivity = 52.8%, specificity = 94.0%, PPV = 21.0%, NPV = 98.5%. For the model without LFTs the respective values were 43.8%, 92.8%, 15.6%, 98.1%.

**Conclusions:**

The Algorithm for Liver Function Investigations (ALFI) is the first model to successfully estimate the probability of all-cause mortality in patients with no apparent liver disease having LFTs in primary care. While LFTs added to the model's discrimination and sensitivity, the clinical utility of ALFI remains to be established since LFTs did not improve an already high NPV for short term mortality and only modestly improved a very low PPV.

## Introduction

Liver function tests (LFTs) are frequently requested and often difficult to interpret in primary care. The results obtained may lead to further invasive and expensive investigations which may be unnecessary. There are a wide variety of reasons for testing liver function including: routine health checks; investigation of non-specific symptoms such as fatigue or nausea; presence of risk factors for liver diseases such as alcohol misuse and/or clinical diagnosis of liver disease; suspected gallbladder or pancreatic problems, and monitoring of drugs such as statins.

Despite increasing numbers of LFTs being performed in the UK [1], [2] some patients continue to present with potentially fatal severe liver disease, which may have been preventable through earlier diagnosis. In patients with raised LFTs but no obvious liver disease, there may be uncertainty about subsequent management. Abnormal LFTs may also signify other serious diseases that might benefit from earlier diagnosis and or intervention (therapeutic and palliative), such as metastatic malignancy, congestive heart failure, and systemic inflammatory conditions [3]–[7]. All of these potential diagnoses add to the uncertainty surrounding management strategies leading to variation in clinical practice with probable over-investigation of some patients and under-investigation of others. In some, early detection and intervention could result in reduced morbidity and mortality and/or better quality of life [8]. Furthermore, patients may have raised LFTs and be asymptomatic which could indicate disease such as non-alcoholic fatty liver disease [2], or they may be healthy and the abnormal result is a false positive. In the latter case the initial abnormality may then lead to unnecessary investigations and secondary care referral causing anxiety and increased health service costs [9]. A decision support tool incorporating a clinical prediction rule could facilitate the management of these patients in primary care.

Clinical prediction models enable accurate probabilities of specific outcomes to be calculated based on characteristics related to the patient, disease or treatment and are a key component of stratified medicine. They are a prognostic strategy often used in primary care [10] of which the Framingham risk score for cardiovascular disease is just one of many [11]–[13]. Before conversion into a user-friendly web-based tool, they must be assessed for predictive ability and externally validated [14], [15]. A model that could predict short-term mortality would enable the GP to identify those patients with a very poor prognosis who need immediate referral to secondary care. It would also identify patients with good prognosis who do not require further investigation.

This population-based historical cohort study followed-up patients living in Tayside, Scotland with no clinically recognised liver disease who initially had LFTs undertaken in primary care [16]. The aim was to derive, assess, and externally validate a predictive model that would estimate the risk of mortality from any cause in liver function tested patients in primary care over a one year period, for subsequent use by general practitioners to aid their decision making.

## Methods

Separate populations were used to develop the prognostic model (derivation cohort) and then externally validate it (validation cohort) [15].

### Derivation cohort

The study population was derived from a population laboratory database which contains all electronically available LFT results from patients within Tayside, Scotland, UK during the fifteen year period from January 1989 to December 2003. Tayside is a mixed urban/rural region characteristic of the UK with a population of approximately 410000 [17]. LFTs included bilirubin, albumin, alkaline phosphatase (ALP), gamma-glutamyltransferase (GGT), alanine transaminase, and aspartate aminotransferase. Since many laboratories only measure either alanine transaminase or aspartate aminotransferase these two similar tests were combined as one test and are referred to as transaminases in the rest of this paper.

**Figure 1:**
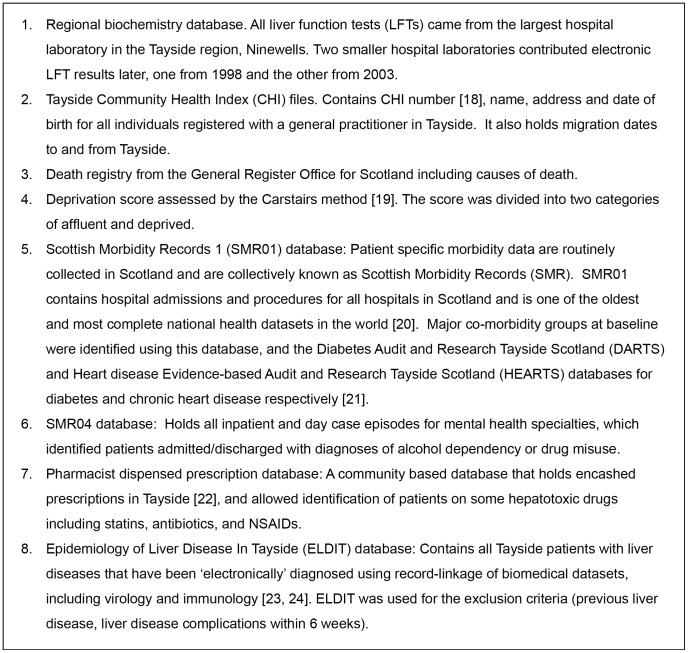
Databases record-linked to create the Tayside derivation cohort.

Patients aged 16 and above with no obvious or reported clinical signs of liver disease upon presentation to a general practitioner, with at least 2 different LFTs requested from the index appointment, between 1989 and 2003 were eligible for inclusion. The following exclusion criteria ensured that the study population of patients had no clinically recognised liver disease at presentation in primary care:

- Patients whose bilirubin result was greater than 35 µmol/L in their initial batch of tests, i.e. clinically jaundiced.
- Patients who had a complication of severe liver disease within 6 weeks of their first LFTs (identified from the ELDIT database detailed in Figure 1). These included ascites, encephalopathy, varices, and portal hypertension.
- Patients with a history of liver disease before the study period (identified from the ELDIT database).

### Databases

In Tayside, all individuals registered with a general practitioner have a unique identifier, the Community Health Index (CHI) [18]. The Health Informatics Centre, University of Dundee, securely hold the CHI files for Tayside. The CHI files contain the CHI number and the patient's name, address and date of birth. The CHI files are used for all health encounters in Tayside and enable record-linkage of both primary and secondary care data. For research studies the datasets listed in Figure 1 were anonymised according to the Standard Operating Procedures of the Health Informatics Centre, University of Dundee [17]. Using the Tayside biochemistry database as the base population, all of the databases listed in Figure 1 were record-linked to it using an anonymous identifier linked to the CHI number.

### Ethics statement

Ethical approval was obtained from the Tayside Committee for Medical Research Ethics in February 2005. Written informed consent from patients was waived by the Tayside Committee for Medical Research Ethics because the databases were anonymised so that no patient identifiable information was accessible. The databases relevant to this study (see Figure 1) covered the entire study period and were used in accordance with procedures approved under the Caldicott Guardian and the Data Protection Act UK (1998) in line with the European directive of 1995.

### Baseline characteristics

The databases (Figure 1) identified baseline characteristics and outcomes of the population. As well as the five LFTs, baseline characteristics included age, gender, deprivation [19], comorbidities (from SMR01) during the period 1980 to study start (including cancer, diabetes, ischaemic heart disease (IHD), stroke, renal disease, respiratory disease, and biliary disease), diagnosed alcohol and drug dependency (from SMR01 and SMR04), methadone use, pregnancy, and use of statins, non-steroidal anti-inflammatory drugs (NSAIDs) or antibiotic use in the three months before LFTs. Since bilirubin was truncated to <36 µmol/L, it was categorised into normal and mildly raised, where normal is <18 µmol/L for males and <16 µmol/L for females.

### Outcomes

The primary outcome was all-cause mortality during the follow-up year. Patients who died were identified from the Scottish National Death Registry. Underlying causes of death were tabulated for the derivation population and categorised using versions 9 and 10 of the International Classification of Diseases.

### Statistical analysis

Parametric survival regression models were fitted to investigate the effect of baseline characteristics on time to all-cause mortality. The starting point was taken as the date of the initial LFT test and the endpoint was one year later, 31^st^ December 2003, date of emigration, or death, whichever was earlier. All patients whose endpoint was not death were censored.

The continuous baseline characteristics of age, albumin, ALP, GGT, and transaminase were assessed for their functional form by plotting them against the Martingale residuals and appropriate transformations were carried out, where necessary. The Weibull accelerated failure time model was used for model building, which was conducted in a manual stepwise manner. A multiple imputation procedure was conducted to impute missing baseline data [25]. Every model during the model building procedure was fitted to 30 imputed datasets arising from the multiple imputation procedure. The parameter estimates and covariances from each imputed dataset were combined to produce inferential results using PROC MIANALYZE in SAS. For each model, the Akaike's information criterion (AIC) statistic was calculated and the average AIC was taken over all 30 imputed datasets. The model with the smallest AIC was considered the optimal model. Two-way covariate interactions were also investigated. The final models were then fitted using different parametric distributions including the generalised gamma, log-logistic, log-normal, and exponential distributions to find the best fit. All patients with complete data were analysed separately as a sensitivity analysis.

The final model was assessed for predictive ability to examine its ability at discriminating high from low risk using the C-statistic [14]. The model's predicted probabilities were assessed for accuracy using calibration plots and testing the calibration slope [26] (more detailed information on multiple imputation and calibration is presented in Appendix S1). The integrated discrimination index (IDI) was used to measure the improvement in the model for each individual covariate [27]. The IDI is the difference between the proportion of variance explained by the full model and the model without the covariate of interest. The sensitivity, specificity, positive predictive value (PPV), and negative predictive value (NPV) were calculated for different risk cut-offs, accounting for censoring [27].

### External validation

The validation cohort contained all patients registered with 19 practices from across Scotland out with Tayside. The practices were participating in the Practice Team Information project operated by the Information Services Division of the National Health Service National Services Scotland, and contributing data to the Primary Care Clinical Informatics Unit, University of Aberdeen [28], [29]. The patient population within the Primary Care Clinical Informatics Unit database is broadly representative of the Scottish population, with respect to age, sex, and social deprivation [29]. The validation cohort contained patients having their initial LFTs measured in primary care between January 2004 and August 2008. All eligible patients had to have test results for ALP, bilirubin, albumin, and transaminase. All baseline characteristics and outcome data obtained for the derivation cohort were also obtained for the validation cohort. No record-linkage to other databases was needed since all of the information was contained in the Primary Care Clinical Informatics Unit database. The same exclusion criteria listed above was also applied to the validation cohort.

The final model was fitted to the validation cohort using the same parameter estimates derived from the study population. The C-statistic was calculated and the calibration plot drawn as for the study population to assess the performance of the model on the external validation cohort. A further model was fitted to the validation cohort using the same covariates as for the final model and its C-statistic computed. The resulting parameter estimates were compared to the final model's parameter estimates using the z-test to test for equality [30]. The calibration slope was tested and the model recalibrated if required.

Analyses were performed using SAS (v9.2) (SAS Institute, Cary, North Carolina).

## Results

### Baseline characteristics

After exclusions our derivation cohort contained 95977 patients with incident initial LFTs taken in primary care and with no obvious liver disease [1]. Only 719 (0.75%) patients had both ALT and AST recorded. In these cases the ALT result was included in the analysis for consistency since the majority of patients with ALT or/and AST tested had ALT tested (87%). Table 1 shows the baseline characteristics of the cohort. There were more females (57.9%) than males (42.1%), and the median (interquartile range) age was 54.6 (39.2–68.8) years. The most frequent previously known baseline co-morbidity was IHD (5.6%), followed by cancer (3.8%). 8.7% of patients were prescribed antibiotics during the three months before their initial LFTs, whilst 3.3% were prescribed statins.

**Table 1:** Baseline and historical characteristics of the derivation cohort (n = 95977) and the validation cohort (n = 11653).

		Cohort n(%) *or* Median (IQR)	
Baseline Characteristics		*Derivation*	*Validation*
Age (years )		54.6 (39.2–68.8)	60.0 (47.0, 72.0)
Gender	Male	40374 (42.1)	5271 (45.2)
	Female	55603 (57.9)	6382 (54.8)
Carstairs category	Affluent	47286 (49.3)	2753 (23.6)
	Deprived	48691 (50.7)	8900 (76.4)
Comorbidity history	Cancer^1^	3629 (3.8)	956 (8.2)
	Diabetes	1386 (1.4)	1441 (12.4)
	IHD	5370 (5.6)	2034 (17.5)
	Renal disease	141 (0.2)	155 (1.3)
	Respiratory disease	2636 (2.8)	883 (7.6)
	Stroke	1471 (1.5)	583 (5.0)
Medication in previous 3 months	Statins	3176 (3.3)	3178 (27.3)
	NSAIDs	6698 (7.0)	1762 (15.1)
	Antibiotics	8307 (8.7)	1962 (16.8)
Abusive substance	Alcohol	2632 (2.7)	465 (4.0)
	Drug	371 (0.4)	0 (0.0)
	Methadone	377 (0.4)	10 (0.1)
Liver function tests	Albumin (g/L)	44.0 (42.0–46.0)	44.0 (41.0, 46.0)
	ALP (U/L)	76.0 (62.0–94.0)	75.0 (62.0, 92.0)
	Transaminase (U/L)	18.0 (14.0–26.0)	21.0 (16.0, 30.0)
	GGT (U/L)	26.0 (17.0–47.0)	27.0 (18.0, 45.0)
	Normal Bilirubin^2^	81111 (91.0)	10587 (90.8)
	Mildly raised Bilirubin^2^	8058 (9.0)	1066 (9.2)

1 Not including biliary cancer or hepatocellular cancer;

2 Normal bilirubin is defined as 0–15 µmol/L for females and 0–17 µmol/L for males; Mildly raised bilirubin is defined as 16–35 µmol/L for females and 18–35 µmol/L for males.

IQR interquartile range; IHD ischaemic heart disease; NSAID non-steroidal anti-inflammatory; ALP alkaline phosphatase; GGT gamma-glutamyltransferase.

### Missing data

Only 8388 (8.7%) patients were tested for all five liver enzymes. The percentage of complete data for each LFT was as follows: ALP (99.2%), albumin (99.2%), bilirubin (93.6%), transaminases (76.5%), and GGT (10.9%). There were more males with complete data (i.e. having all five liver enzymes measured) than females (54.6% versus 45.4%) (Appendix S2). The group with complete data were also more deprived and contained more alcohol dependent patients than the incomplete data group. A multiple imputation procedure was performed to impute missing or untested LFT results as detailed in appendix S1.

### All-cause mortality

A total of 2613 patients (2.7%) died of any cause during one year follow-up. The commonest underlying cause of death was cancer (39.3%) (Table 2). Of these, gastrointestinal (30.6%) and lung cancers (29.7%) were the most frequent. Of those who died from cancer, 77% had no history of cancer recorded at baseline. Diseases of the circulatory system were the second commonest cause of death (34.7%), and of these, IHD was the most frequent (55.9%). Of those who died from IHD, 77% had no history of IHD. Cause of death was missing for 90 patients (3.4%).

**Table 2:** Causes of mortality within one year of liver function tests.

Underlying cause of death	n	%
Neoplasms:	1027	39.3
*Malignant neoplasm of digestive organs and peritoneum*	*314*	*12.0*
*Malignant neoplasm of respiratory and intrathoracic organs*	*305*	*11.7*
*Malignant neoplasm of other and unspecified sites*	*138*	*5.3*
*Malignant neoplasm of genitourinary organs*	*125*	*4.8*
*Malignant neoplasm of bone, connective tissue, skin and breast*	*72*	*2.8*
*Malignant neoplasm of lymphatic and hematopoietic tissue*	*49*	*1.9*
*Malignant neoplasm of lip, oral cavity and pharynx*	*13*	*0.5*
*Neoplasm of unspecified nature*	*6*	*0.2*
*Neoplasm of uncertain behaviour*	*2*	*0.1*
*Benign neoplasm*	*2*	*0.1*
*Malignant neoplasm of independent (primary) multiple sites*	*1*	*0.0*
Diseases of the circulatory system:	907	34.7
*Ischaemic heart disease*	*507*	*19.4*
*Cerebrovascular disease*	*208*	*8.0*
*Other forms of heart disease*	*94*	*3.6*
*Diseases of arteries, arterioles, and capillaries*	*36*	*1.4*
*Diseases of pulmonary circulation*	*26*	*1.0*
*Hypertensive disease*	*15*	*0.6*
*Diseases of veins and lymphatics, and other diseases of circulatory system*	*13*	*0.5*
*Chronic rheumatic heart disease*	*8*	*0.3*
Diseases of the respiratory system:	258	9.9
*Pneumonia and influenza*	*119*	*4.6*
*Chronic obstructive pulmonary disease and allied conditions*	*103*	*3.9*
*Other diseases of respiratory system*	*26*	*1.0*
*Pneumoconioses and other lung diseases due to external agents*	*9*	*0.3*
*Acute respiratory infections*	*1*	*0.0*
Diseases of the digestive system	62	2.4
Injury and poisoning	59	2.3
Mental disorders	57	2.2
Diseases of the nervous system and sense organs	45	1.7
Diseases of the genitourinary system	39	1.5
Endocrine, nutritional and metabolic diseases, and immunity disorders	23	0.9
Diseases of the musculoskeletal system and connective tissue	14	0.5
Certain infectious and parasitic diseases	12	0.5
Diseases of blood and blood forming organs	8	0.3
Symptoms, signs and ill-defined conditions	8	0.3
Diseases of the skin and subcutaneous tissue	3	0.1
Congenital malformations, deformations and chromosomal abnormalities	1	0.0
Missing	90	3.4
Total	2613	100.0

Note: Causes of death in italics are subsets of those in normal font.

### Prediction of all-cause mortality

The final model is presented in Table 3 where the baseline characteristics are sorted in descending order of the IDI statistic. All five LFTs were predictive of mortality with albumin the strongest, followed closely by ALP. History of cancer had a strong effect on mortality, and renal disease, stroke, IHD, and respiratory disease were also highly associated with mortality. The model also indicated that being male, increasing age and deprivation were associated with increased risk of death within a year. Statins were significantly associated with reduced risk of mortality. Age interacted with gender, deprivation, cancer, ALP, and transaminase, and although these were significant terms, the coefficients were small. History of diabetes, NSAID use, antibiotic use, methadone use, alcohol dependency, and drug dependency were not predictive of mortality. The IDI statistic showed that patient age explained the greatest percentage of variance in the model. This was followed by albumin and ALP result. Adding the four LFTs to the model without any LFTs gave an IDI of 15%.

**Table 3:** Parameter estimates of the final generalised gamma model predicting risk of all-cause mortality within 1 year of initial liver function tests.

Parameter	Coefficient (95% CI)	P-value	IDI, %
Intercept	15.158 (12.414 to 17.902)	<0.001	
Age at baseline	−0.127 (−0.163 to −0.091)	<0.001	8.43^a^
Albumin	0.194 (0.181 to 0.207)	<0.001	4.66
Log (ALP)	−2.179 (−2.767 to −1.592)	<0.001	1.24^a^
Cancer (yes *v* no)	−4.538 (−5.393 to −3.683)	<0.001	0.42^a^
Gender (Male *v* Female)	−1.184 (−1.734 to −0.634)	<0.001	0.33^a^
Log (transaminase)	1.323 (0.755 to 1.891)	<0.001	0.26^a^
Log (GGT)	−0.453 (−0.660 to −0.246)	<0.001	0.17
Stroke (yes *v* no)	−0.587 (−0.800 to −0.375)	<0.001	0.13
Respiratory disease (yes *v* no)	−0.605 (−0.794 to −0.417)	<0.001	0.11
Bilirubin (mildly raised *v* normal)	−0.403 (−0.553 to −0.253)	<0.001	0.11
Renal disease (yes *v* no)	−0.969 (−1.520 to −0.418)	<0.001	0.09
Statins (yes *v* no)	0.617 (0.320 to 0.913)	<0.001	0.07
Deprived (yes *v* no)	−0.999 (−1.535 to −0.462)	<0.001	0.07^a^
IHD (yes *v* no)	−0.259 (−0.409 to −0.110)	<0.001	0.05
Age at baseline X Cancer	0.050 (0.038 to 0.061)	<0.001	
Age at baseline X Log (ALP)	0.017 (0.009 to 0.025)	<0.001	
Age at baseline X Deprived	0.011 (0.004 to 0.018)	0.002	
Age at baseline X Log (transaminase)	−0.010 (−0.017 to −0.003)	0.006	
Gender X Age at baseline	0.010 (0.003 to 0.018)	0.007	
*Scale*	*1.762 (1.625 to 1.899)*	*<0.001*	
*Shape*	*0.408 (0.314 to 0.502)*	*<0.001*	

a the relative interaction terms containing this parameter were also excluded.

IHD = ischaemic heart disease; GGT = gamma-glutamyltransferase; ALP = alkaline phosphatase; IDI = Integrated Discrimination Improvement.

Notes: The baseline parameters are listed in decreasing order of IDI. A negative coefficient for a parameter indicates an increasing effect on mortality risk, whilst a positive coefficient indicates a decreasing effect on mortality risk.

### Predictive ability of derivation cohort model

**Figure 2:**
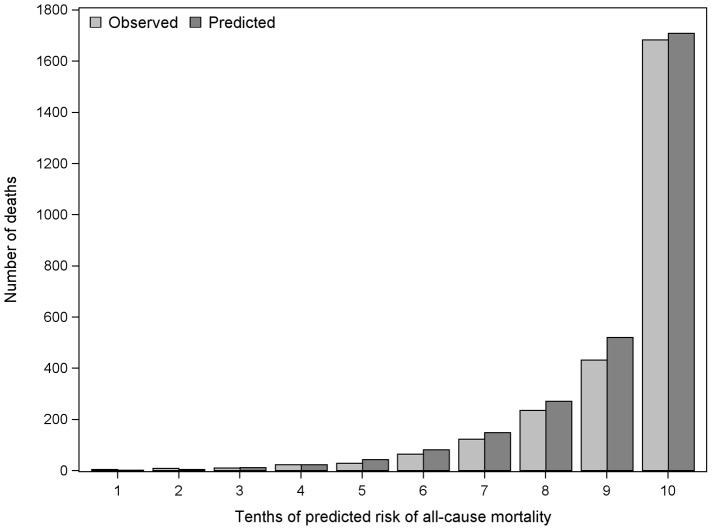
Number of predicted and observed mortality events one year after initial LFTs.

The C-statistic for discriminatory ability of the prediction model for risk of one year mortality was 0.82 (95% CI 0.80, 0.84). With LFTs excluded the C-statistic was lower with a value of 0.79 (95% CI 0.76, 0.81) in the derivation cohort, and demonstrates that LFTs add some discriminatory ability to the model. Figure 2 displays the observed versus the predicted number of deaths by tenths of predicted probability of mortality from the model. Although tenths 5 to 9 show some visible evidence of over-prediction of mortality, the top tenth of predicted mortality is similar to the observed mortality. The calibration slope test showed no evidence of over-fitting (see Appendix S3). The sensitivity, specificity, PPV and NPV for different cut-offs of predicted risk of mortality are displayed in Table 4. For example, a cut-off greater than or equal to 0.61% (the median predicted probability of mortality) had a low PPV of 5.7% and high NPV of 99.8%.

**Table 4:** Performance measures for different cut-offs of predicted probability of mortality for the final model.

Cut-off for predicted probability,%	Number (%) of patients over cut-off (out of 95,977)	Sensitivity, %	Specificity, %	PPV, %	NPV, %
≥0.611^a^	47989 (50)	97.0	51.4	5.69	99.8
≥2.78^b^	23996 (25)	84.8	76.8	9.94	99.4
≥5	15068 (15.70)	72.9	86.0	13.6	99.1
≥7.75^c^	9598 (10)	60.7	91.5	17.8	98.7
≥10	7058 (7.35)	52.8	94.0	21.0	98.5
≥13.2^d^	4799 (5)	43.6	96.2	25.6	98.3
≥20	2546 (2.65)	30.7	98.2	33.9	97.9
≥30	1173 (1.22)	18.9	99.3	45.3	97.6
≥32.9^e^	960 (1)	16.5	99.5	48.4	97.5
≥40	620 (0.65)	12.2	99.7	55.1	97.4
≥50	323 (0.34)	7.47	99.9	64.9	97.3
≥60	173 (0.18)	4.55	100	73.6	97.2
≥70	96 (0.10)	2.79	100	81.0	97.1

Cut-offs at: ^a^50th; ^b^75th; ^c^90th; ^d^95th; ^e^99th percentile.

PPV = positive predictive value; NPV = negative predictive value.

For a model without LFTs the performance measures were similar to the full model for the median cut-off (see Table 5). For example, for a cut-off greater than or equal to 10%, the PPV increased from 15.6% to 21.0% for the full model. The sensitivity, specificity and NPV increased from 43.8% to 52.3%, from 92.8% to 94.0%, and from 98.1% to 98.5% respectively.

**Table 5:** Performance measures for different cut-offs of predicted probability of mortality for the final model excluding all LFT terms.

Cut-off for predicted probability,%	Number (%) of patients over cut-off (out of 95,977)	Sensitivity, %	Specificity, %	PPV, %	NPV, %
≥0.953^a^	47990 (50)	95.4	51.4	5.77	99.7
≥3.84^b^	23994 (25)	78.1	76.7	9.45	99.1
≥5	19235 (20.04)	71.1	81.6	10.7	98.9
≥8.98^c^	9598 (10)	48.6	91.2	14.7	98.3
≥10	8031 (8.37)	43.8	92.8	15.6	98.1
≥13.0^d^	4798 (5)	30.7	95.8	18.6	97.8
≥20	1317 (1.37)	12.5	99.0	25.6	97.3
≥21.8^e^	959 (1)	9.28	99.3	28.0	97.2
≥30	257 (0.27)	4.22	99.9	34.5	97.0
≥40	54 (0.06)	1.55	100.0	41.2	97.0
≥50	8 (0.01)	0.289	100.0	49.4	97.0
≥60	3 (0.003)	0.123	100.0	51.1	97.0

Cut-offs at: ^a^50th; ^b^75th; ^c^90th; ^d^95th; ^e^99th percentile.

PPV = positive predictive value; NPV = negative predictive value.

### External validation

The external cohort contained 11653 patients (Table 1). The proportion of males and females were reasonably similar to the population used to develop the model (45.2% versus 42.1% males). The median age was five years older and there were a greater proportion of deprived patients (76.4% versus 50.7%). There were also consistently more patients with co-morbidities and medications. However, the median LFTs were similar between the two cohorts. GGT was missing for 4178 (35.9%) patients and so were imputed in a similar manner as for the main study population. A total of 325 patients (2.8%) died within one year.

The C-statistic for the final model applied to the external cohort was 0.86 (95% CI 0.79 to 0.90). The calibration slope test was borderline significant meaning that the model required a small amount of recalibrating which is usual in external validation (more detail in Appendix S4).

### Survival curves for specific groups and cases

**Figure 3:**
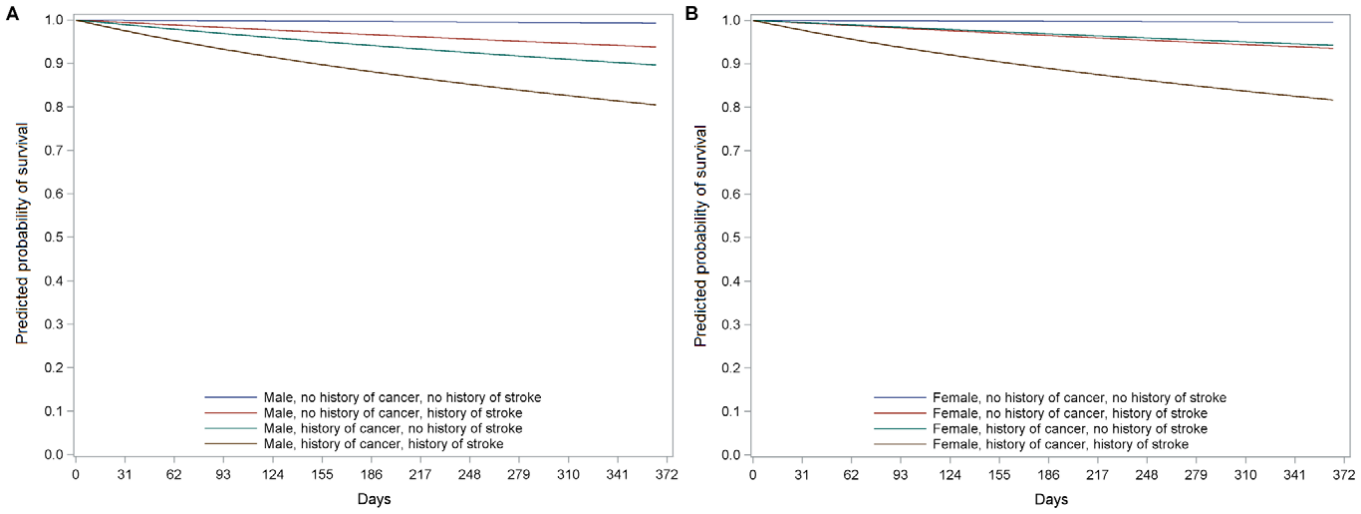
Survival curves for males (a) and females (b) by history of cancer and stroke status during the first year of follow-up.

**Figure 4:**
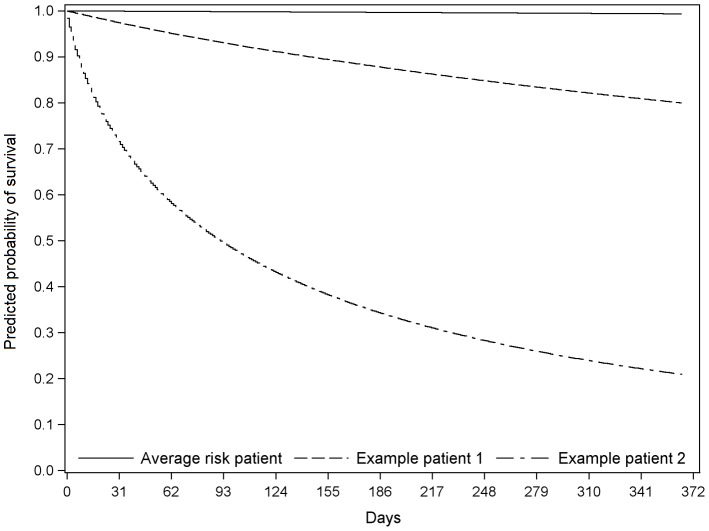
Predicted probability of surviving during the year for the average risk patient and two example cases. Example 1 patient: 55 year old male, albumin = 28 g/L, ALP = 137 U/L, bilirubin = 9 µmol/L, GGT = 86 U/L and transaminase = 41 U/L. Example 2 patient: 54 year old male, history of cancer, albumin = 38 g/L, ALP = 1133 U/L, bilirubin = 8 µmol/L, GGT = 114 U/L and transaminase = 25 U/L. Probability of surviving up to one year for each of these patients was 0.99, 0.80, and 0.21 respectively.

Figure 3 presents the survival curves for males (Figure 3a) and females (Figure 3b) with different histories of cancer and stroke during the first year of follow-up. Although the curves are quite similar for males and females, males had lower survival probabilities, especially those with a history of stroke. Figure 4 shows the probability of survival during the year for the average risk patient, a specific moderate-high risk case, and a specific very high risk case (the characteristics of these latter two patients are as described in the legend of Figure 4). The formula for calculating mortality risk is presented in Appendix S5.

## Discussion

We have developed and validated a model, the Algorithm for Liver Function Investigations (ALFI), that can predict mortality within one-year following liver function testing in primary care. ALFI performs better at lower cut-offs of predicted probabilities. The NPV and specificity measures were excellent but decreased little when LFTs were removed from the model (for NPV this reflects the low overall risk of mortality). However, whilst LFTs did improve sensitivity and moderately improved an already low PPV, the clinical utility needs to be established, e.g. through the use of a web-based tool to see the impact of ALFI on clinical decision making, further investigations, patient outcomes, and costs to the health service. The majority of deaths were from previously undiagnosed cancer and cardiac disease which may warrant further investigation.

ALFI will give an individualised prognosis based on the patient's characteristics, LFTs, comorbidity history and statin use. The resulting probability estimate combined with other clinical information (not available as potential predictors for the model) at the GP's disposal might facilitate their decision-making with regards to further investigations, referral or watchful waiting. With its extremely high NPVs, ALFI has an excellent ability to accurately assign a low probability of short term mortality. For example, if we take 0.61% as our predicted probability cut-off, the model can accurately identify those patients with a very low probability of poor prognosis and the GP can confidently use this with any other contextual information not in the model e.g. body mass index, smoking status, alcohol intake, to inform their decision making. Using the model alone, with such a cut-off the GP would under investigate 83/47989 (0.2%) patients but correctly rule-out one-year mortality in 47906/47989 (99.8%) patients. Furthermore, at this cut-off the sensitivity is very high since ALFI detected 97% of all actual deaths within the year. However, the PPV was low meaning that 94.3% of patients detected over the cut-off would be over-investigated. However patient outcomes beyond one year may be improved by investigation. The use of clinical parameters without LFTs in the model had high enough sensitivity to give very similar NPVs given the low overall risk of mortality. However, the PPV increased at a lesser rate and sensitivity reduced at a greater rate as the cut-offs increased meaning that LFTs contributed more to the proportion of true positives than to true negatives. For example, for a predicted probability cut-off at the 75^th^ percentile (3.84% for the model without LFTs and 2.78% for the model with LFTs) the sensitivity improved from 78.1% without LFTs to 84.8% with LFTs. Even with LFTs included the PPV never gets as high as the NPV and as it increases the sensitivity decreases dramatically. For example, if we used the model to refer those with a very high mortality risk of greater than 60%, the GP would over-investigate 26.4% patients but correctly investigate 73.6% patients. However, at this cut-off the sensitivity is extremely poor meaning that ALFI would fail to detect 95.4% of those who did die within the year. With such small numbers at high cut-offs and with poor sensitivity, it is clear that ALFI performs best at low cut-offs of predicted risk but at the expense of low PPV.

One prediction model involving liver disease is the Model for End-Stage Liver Disease (MELD) [31]. This model was originally developed to predict short-term survival (3 months and one year) of patients with cirrhosis who were about to go through the transjugular intrahepatic portosystemic shunt procedure. MELD consisted of a range of parameters including serum bilirubin, creatinine levels, International Normalised Ratio for prothrombin time, and aetiology of liver disease. In 2001, the model was validated for a wider group of patients, with a range of liver disease severity and aetiology, who were awaiting liver transplant [32]. MELD was then developed as a replacement for the use of waiting time as a measure of the organ allocation priority.

Similarly, the ALFI model could be used to prioritise referrals to secondary care. However, it would not tell the user who to refer to as this would require additional contextual information and clinical judgement. ALFI's main use could be to identify those who have a very low probability of death and do not need further investigation for severe life threatening conditions. It would not, however, detect those who have important underlying conditions that might reduce survival in the longer term or non fatal conditions than can impair quality of life and still need a diagnosis and appropriate treatment.

### Strengths and weaknesses

This is the first successfully derived and externally validated population risk prediction model for one year all-cause mortality in primary care patients with LFTs using a large population dataset. The ability of the model to discriminate between patients at high and low risk was excellent (c = 0.82) and the calibration curve showed reasonable accuracy of the probability estimates from the prediction model across the range of values of predicted risk. The discrimination of ALFI when applied to the external cohort was also excellent (c = 0.86). In comparison, the Framingham equation c-statistic ranged from 0.63 to 0.83 for its external validation on six different cohorts [30], and a model predicting risk of emergency admissions reported a discrimination of 0.79 for its validation group [13].

ALFI was derived from unselected “real-world” observations in a geographically defined population: an approach being encouraged by the National Institutes of Health [33]. Strengths of the data used in this study were the large study population size, the high quality of established national databases, and the deterministic linkage using the CHI number. A weakness of electronic databases was the lack of some potentially useful predictors of mortality, such as alcohol intake, smoking, presence or severity of heart failure, and body mass index. Whilst we have no specific data on the clinical indications for requesting LFTs, we were able to identify patients with a history of major co-morbidities since 1980, including cancer, diabetes, and IHD using SMR hospital admission records and population registers, and patients who were prescribed statins, NSAIDs, and antibiotics in the three months before their initial LFTs. Although patients with a diagnosed history of liver disease were excluded from the analysis, it is possible that patients with very early asymptomatic undiagnosed liver disease were included. However, we have no way of estimating this from the data. In fact, one could argue that clinical indications, asymptomatic undiagnosed liver disease, alcohol dependency and obesity may correlate highly with LFTs, since the latter are markers for such [3], [34], [35], resulting in multicollinearity and their exclusion during the model building process. As for all statistical models, residual confounding may be present due to not adjusting for unavailable factors such as drugs. For example, metformin which is used to treat Type 2 diabetes has been shown to prevent cancers in the digestive organs [36], [37]. With the recent advancement of primary care data extraction systems [38], the potential for incorporating further databases e.g. genetic [39], and the improvement in data recording, we will have the ability in the future to include further potential prognostic factors to update ALFI.

LFTs are non-specific markers of illness i.e. either disease processes in the liver, local involvement from disorders of other organs, or as a marker of systemic disease. The inflammatory or infective liver diseases tend to produce abnormality of the transaminases and may produce mortality rises in the long term (decades) but the effects of other organ disorders (e.g. aggressive cancers or heart disease) are more likely to be manifest in the short term, so a one-year follow-up period was chosen for this study. The algorithm is not a diagnostic one but an aid to clinical decision making and all-cause mortality was deemed the most appropriate to ‘catch all’ patients with very poor or very good prognosis. Clearly, a model for mortality over a longer follow-up period would have a poorer predictive ability following one set of LFTs since other events would intervene. In contradistinction the use of LFTs as a predictor of outcome in chronic liver disease would need a much longer follow-up period as the natural history of most primary liver diseases is decades. The model including LFTs had better discriminatory ability and higher PPV than the model without. Furthermore, albumin and ALP explained the second and third most variation of all the predictors in the model proving that LFTs are valuable prognostic factors for short term mortality.

### GGT

GGT was missing for a large proportion of patients in the derivation cohort but less so in the validation cohort. However the appropriate guidelines for handling this problem were followed [40]. The demographics of the patients with complete data (i.e. males, illicit drug users, alcohol dependents, and patients living in deprived areas) suggested that general practitioners requested GGT where they suspected that there may be a chance of substance abuse [41]. Therefore it was assumed that the missing data depended on variables in the observed data - the missing at random assumption which is required for multiple imputation [25]. Although the percentage of patients untested for GGT was 89.1%, with such a large cohort this still meant that data from 10484 patients were used to impute GGT. Relative efficiency is the efficiency of an estimate obtained from *m* imputations relative to one obtained from an infinite number of imputations [25]. The value used is arbitrary but obviously the closer to 100% the better. The dataset was imputed 30 times to allow a more precise parameter estimate for GGT which gave a relative efficiency value of 97.1% for 90% missing data. When the final model was fitted to a complete dataset excluding GGT the parameter estimates were reasonably similar to those from the model fitted to the imputed dataset. This is reassuring as it suggests that the inclusion of a highly imputed GGT does not cause the other predictors parameter estimates to vary by much, thus ruling out biased imputations of GGT (Appendix S2). In Tayside, the laboratories do not routinely include GGT with the other four LFT results unless specifically requested by the general practitioner. However, in the external validation cohort GGT was much more complete (64.1%) meaning that other labs do measure GGT routinely. Therefore we perceive the inclusion of GGT in the model as an advantage so that GPs from these regions can include this test in the model. We have shown that GGT had the seventh highest IDI value of all the predictors, suggesting that its use should be re-evaluated in those regions that do not test for it consistently.

### Cancer

Out of 95977 patients from the derivation cohort, 2613 died (2.7%) within one year. Almost 40% of the 2613 deaths were caused by cancer. A Korean study prospectively followed up 142055 men and women who had a transaminase test for a maximum period of 8 years and they also found that out of 3786 deaths, 46.2% were from cancer [6]. A history of cancer was also highly predictive of mortality within one year. Over three-quarters of those who died from cancer did not have a history of the disease. Further research could involve determining the predictive ability of LFTs for outcomes related to cancer in those without known cancer diagnosis.

### External validation

External validation of prediction models is extremely important in order to support their application and transportability to different geographical or temporal populations. Since our external cohort came from 19 primary care practices across nine different regions of Scotland during a different time period than the population used to develop the model, we have shown that ALFI predicts well with regards to both aspects. The fact that the external cohort had patients with greater rates of deprivation and comorbidity than the derivation cohort shows that the model is robust to changes in baseline characteristics. The median LFT values for both cohorts were very similar, as were the proportions of patients who died within one-year. The next step is to convert ALFI into a web-based decision aid to assess its impact on GP management of these patients. Using the sensitivity and PPV results, suggested management plans can be determined for different risk cut-offs. A feasibility study of its implementation, focused on general practitioner acceptability and impact on decision making and costs might then usefully follow.

### Conclusions

This study has developed and externally validated a novel risk prediction model for one-year mortality in patients, with no clinically obvious liver disease, having their LFTs taken in primary care. All five LFTs were predictive of mortality and improved the discriminatory ability, sensitivity and PPV of the model. ALFI performs best at lower cut-offs of predicted probabilities of mortality with excellent sensitivity and NPV and good specificity. However, low PPV may mean over-investigation of some patients. However, the addition of LFTs did not improve NPV for short term mortality in these types of patient and only modestly improved the PPV. Therefore the clinical utility of ALFI as a decision tool in primary care needs to be established in terms of further testing, patient outcomes and health service costs. The utility of liver function testing per se needs to be further examined using other outcomes such as impact on the detection of modifiable diseases such as chronic liver disease.

## Supporting Information

Appendix S1 **Further information on statistical methods.**
(DOC)

Appendix S2 **Complete case analyses.**
(DOC)

Appendix S3 **Calibration of the final model.**
(DOC)

Appendix S4 **Discrimination and calibration of the model applied to the external cohort.**
(DOC)

Appendix S5 **How to calculate the predicted probability of mortality within T days (where 1≤T≤365).**
(DOC)
