# Supplementary material for: The Utility of Liver Function Tests for Mortality Prediction within One Year in Primary Care Using the Algorithm for Liver Function Investigations (ALFI)
Source: PLoS One. 2012 Dec 14;7(12):e50965. doi: 10.1371/journal.pone.0050965 (PMC3522690; doi:10.1371/journal.pone.0050965)
Supplement: Appendix S1 — Further information on statistical methods. (DOC) [file pone.0050965.s001.doc]

**Appendix S1**

Further information on statistical methods

*Missing data*

The problem of missing data occurs in almost all retrospective studies using routine health databases. The simplest way of dealing with this is to use only cases with complete data in the analysis. However, this leads to the loss of potentially valuable information from the incomplete cases (and hence loss of power), and introduces bias, especially if there are systematic differences between the complete and incomplete cases. Therefore, the findings from an analysis using only the complete cases may not be a true reflection on what would be found if all the cases were analysed. To analyse only the complete data assumes that the missing data are missing completely at random (MCAR) which is unlikely [1,2]. A weaker version of the MCAR assumption is the missing at random (MAR) assumption. This differs from MCAR in that it assumes the missing data are missing at random but can depend on one or more variables in the observed data [1].

Assuming MAR, a multiple imputation technique using the Markov Chain Monte Carlo (MCMC) method was used to impute the missing values [3]. The multiple imputation procedure in SAS (PROC MI) was used to impute 30 datasets with each imputation made after every 500 iterations. A log transformation was applied to skewed continuous variables to make them normally distributed. All baseline characteristics, time to death (or censored event) and death outcome were included in the multiple imputation procedure. The complete data were also analysed separately as a sensitivity analysis.

***Model performance***

The final model was assessed for predictive ability to examine its ability at discriminating high from low risk using the C-statistic [4]. The model’s predicted probabilities were assessed for accuracy using calibration plots and tests. Calibration assesses the accuracy of the probability estimate from the prediction model across the range of values of predicted risk. The cohort was divided into intervals of increasing risk with each interval containing 50 patients, and the mean predicted probability of survival calculated for each. The mean actual probability of survival using the Kaplan-Meier method was also calculated in each interval. The mean predicted probabilities were then plotted against the mean actual probabilities. A model with perfect calibration would see the points lie along the diagonal. A non-parametric calibration curve was fitted to the points using a Loess smoothing curve [5].

A calibration slope indicates how much of a reduction of the effects of predictors is needed on average to ensure that the model is well calibrated for new patients from the underlying population [6]. The calibration slope (b_slope_) was calculated by fitting the linear predictor (Xβ) for the patients used to develop the final model in a separate model by itself i.e. Y=a + b_slope_ *Xβ. Depending on the AFT model used, any scale or shape parameters were held fixed at their values for the final model. If the slope of the Xβ (i.e. b_slope_) deviated significantly from 1 (found by fitting the same model including Xβ as an offset and testing the regression coefficient of Xβ, now the slope deviance b_dev_, using the Wald test) then the final model would need adjusted for this ‘over-fitting’ of predictors or miscalibration by including the intercept and deviation (a + b_dev_ *Xβ) in the model as a covariate.

All model performance procedures were performed on a dataset with average values taken over the 30 imputed datasets for each of the imputed variables for each patient.

**References**

1. Yuan YC. Multiple imputation for missing data: concepts and new development: SAS Institute Inc.

2. Rubin DB (1976) Inference and missing data. Biometrika 63: 581–592.

3. Rubin DB (1987) Multiple imputation for nonresponse in surveys. New York: Wiley.

4. Pencina MJ, D'Agostino RB (2004) Overall C as a measure of discrimination in survival analysis: model specific population value and confidence interval estimation. Stat Med 23: 2109–2123.

5. Harrell FE, Lee KL, Mark DB (1996) Multivariable prognostic models: issues in developing models, evaluating assumptions and adequacy, and measuring and reducing errors. Stat Med 15: 361–387.

6. Steyerberg EW (2009) Clinical prediction models: a practical approach to development, validation, and updating. New York: Springer.
