# Supplementary material for: The Utility of Liver Function Tests for Mortality Prediction within One Year in Primary Care Using the Algorithm for Liver Function Investigations (ALFI)
Source: PLoS One. 2012 Dec 14;7(12):e50965. doi: 10.1371/journal.pone.0050965 (PMC3522690; doi:10.1371/journal.pone.0050965)
Supplement: Appendix S2 — Complete case analyses. (DOC) [file pone.0050965.s002.doc]

Appendix S2

Complete case analyses

The final model was fitted to two complete data subgroups to compare with the imputed data results. The first was the fully complete dataset containing 8388 patients who had all five LFTs measured (see Table S1 for baseline characteristics). Many baseline characteristics were not statistically significant in this model (see Table S2). The only significant covariates were age, GGT, albumin, transaminase, ALP, age X ALP, and age X transaminase. The second data subgroup contained only patients who had all four of transaminase, albumin, bilirubin, and ALP tested (n=68852). In other words GGT was excluded from the model entirely. The parameter estimates of this model were reasonably similar to those of the model derived from the imputed dataset (Table S3). The only predictor that was not statistically significant was history of IHD (p=0.16).

Table S1 Comparison of baseline characteristics in patients with complete data with patients with incomplete data

| **Baseline Characteristics** | | | **Complete data**  **(n=8388)** | **Incomplete data**  **(n=87589)** |
| --- | --- | --- | --- | --- |
| Age (years ) | | | 52.0 (39.3-65.1) | 55.0 (39.2-69.1) |
| Gender | Male | | 4578 (54.6) | 35796 (40.9) |
|  | Female | | 3810 (45.4) | 51793 (59.1) |
| Carstairs category | Affluent | | 3316 (39.5) | 43970 (50.2) |
|  | Deprived | | 5072 (60.5) | 43619 (49.8) |
| Comorbidity | Cancer^1^ | | 316 (3.8) | 3313 (3.8) |
|  | Diabetes | | 78 (0.9) | 1308 (1.5) |
|  | IHD | | 381 (4.5) | 4989 (5.7) |
|  | Renal disease | | 10 (0.1) | 131 (0.2) |
|  | Respiratory disease | | 198 (2.4) | 2438 (2.8) |
|  | Stroke | | 107 (1.3) | 1364 (1.6) |
| Medication in previous 3 months | Statins | | 182 (2.2) | 2994 (3.4) |
|  | NSAIDs | | 415 (5.0) | 6283 (7.2) |
|  | Antibiotics | | 755 (9.0) | 7552 (8.6) |
| Abusive substance | Alcohol | | 534 (6.4) | 2098 (2.4) |
|  | Drug | | 49 (0.6) | 322 (0.4) |
|  | Methadone | | 43 (0.5) | 334 (0.4) |
| Liver function tests | Albumin (g/L) | | 44.0 (41.0-46.0) | 44.0 (42.0-46.0) |
|  | ALP (U/L) | | 75.5 (61.0-95.0) | 76.0 (62.0-94.0) |
|  | Transaminase (U/L) | | 20.0 (15.0-30.0) | 18.0 (14.0-25.0) |
|  | GGT (U/L) | | 26.0 (17.0-48.0) | 26.0 (17.0-46.0) |
|  | Bilirubin^2^ | |  |  |
|  |  | Normal | 7447 (88.8) | 73664 (91.2) |
|  |  | Mildly raised | 941 (11.2) | 7117 (8.8) |
| Follow-up time |  | | 365 (365-365) | 365 (365-365) |
| Died within 1 year |  | | 286 (3.4) | 2327 (2.7) |

Data reported are median (interquartile range) or percentage

^1^Not including biliary cancer or hepatocellular cancer;

^2^Normal bilirubin is defined as 0-15μmol/L for females and 0-17μmol/L for males; Mildly raised bilirubin is defined as 16-35μmol/L for females and 18-35μmol/L for males.

IHD ischaemic heart disease; NSAID non-steroidal anti-inflammatory; ALP alkaline phosphatase; GGT gamma-glutamyl transferase

Table S2 Model predicting risk of all-cause mortality within 1 year of initial liver function tests for patients with complete data only (N=8388)

| **Parameter** | **Coefficient (95% CI)** | **P-value** |
| --- | --- | --- |
| Intercept | 16.174 (9.614 to 22.735) | <0.001 |
| Albumin | 0.176 (0.140 to 0.211) | <0.001 |
| Log (ALP) | -2.956 (-4.291 to -1.622) | <0.001 |
| Log (GGT) | -0.441 (-0.642 to -0.240) | <0.001 |
| Log (transaminase) | 1.978 (0.857 to 3.100) | <0.001 |
| Age at baseline X Log (ALP) | 0.030 (0.011 to 0.048) | 0.002 |
| Age at baseline | -0.134 (-0.222 to -0.045) | 0.003 |
| Age at baseline X Log (transaminase) | -0.023 (-0.038 to -0.008) | 0.004 |
| Cancer (yes vs. no) | -2.563 (-5.230 to 0.105) | 0.06 |
| Renal disease (yes vs. no) | -1.350 (-2.987 to 0.287) | 0.11 |
| Statins (yes vs. no) | 0.894 (-0.283 to 2.071) | 0.14 |
| Age at baseline X Deprived | 0.012 (-0.009 to 0.033) | 0.25 |
| Deprived (yes vs. no) | -0.851 (-2.336 to 0.634) | 0.26 |
| Age at baseline X Cancer | 0.020 (-0.016 to 0.057) | 0.27 |
| Bilirubin (mildly raised vs normal) | -0.151 (-0.567 to 0.264) | 0.48 |
| Stroke (yes vs. no) | -0.269 (-1.026 to 0.489) | 0.49 |
| Respiratory disease (yes vs. no) | -0.173 (-0.859 to 0.512) | 0.62 |
| Gender (Male vs. Female) | -0.316 (-1.778 to 1.146) | 0.67 |
| IHD (yes vs. no) | -0.067 (-0.577 to 0.442) | 0.80 |
| Gender X Age at baseline | 0.001 (-0.020 to 0.021) | 0.94 |
| Scale | 1.835 (1.485 to 2.268) |  |
| Shape | 0.326 (0.038 to 0.614) |  |

IHD ischaemic heart disease; GGT = gamma-glutamyl transferase; ALP = alkaline phosphatase

Table S3 Model predicting risk of all-cause mortality within 1 year of initial liver function tests for patients with complete data only, excluding GGT (N=68852)

| **Parameter** | **Coefficient (95% CI)** | **P-value** |
| --- | --- | --- |
| Intercept | 15.789 (12.671 to 18.908) | <0.001 |
| Albumin | 0.206 (0.190 to 0.222) | <0.001 |
| Cancer (yes vs. no) | -4.617 (-5.617 to -3.616) | <0.001 |
| Log (ALP) | -2.568 (-3.224 to -1.912) | <0.001 |
| Age at baseline X Cancer | 0.051 (0.037 to 0.064) | <0.001 |
| Age at baseline | -0.138 (-0.179 to -0.098) | <0.001 |
| Stroke (yes vs. no) | -0.653 (-0.904 to -0.402) | <0.001 |
| Bilirubin (mildly raised vs normal) | -0.411 (-0.583 to -0.239) | <0.001 |
| Age at baseline X Log (ALP) | 0.021 (0.012 to 0.030) | <0.001 |
| Respiratory disease (yes vs. no) | -0.493 (-0.728 to -0.259) | <0.001 |
| Renal disease (yes vs. no) | -1.260 (-1.873 to -0.647) | <0.001 |
| Log (transaminase) | 1.010 (0.473 to 1.547) | <0.001 |
| Statins (yes vs. no) | 0.550 (0.240 to 0.860) | <0.001 |
| Gender (Male vs. Female) | -1.162 (-1.819 to -0.506) | <0.001 |
| Deprived (yes vs. no) | -1.109 (-1.751 to -0.466) | <0.001 |
| Age at baseline X Log (transaminase) | -0.012 (-0.019 to -0.004) | 0.002 |
| Age at baseline X Deprived | 0.014 (0.005 to 0.022) | 0.002 |
| Gender X Age at baseline | 0.009 (0.0001 to 0.018) | 0.048 |
| IHD (yes vs. no) | -0.132 (-0.314 to 0.051) | 0.16 |
| Scale | 1.707 (1.550 to 1.879) |  |
| Shape | 0.462 (0.350 to 0.574) |  |

IHD ischaemic heart disease; GGT = gamma-glutamyl transferase; ALP = alkaline phosphatase
