# Supplementary material for: The Utility of Liver Function Tests for Mortality Prediction within One Year in Primary Care Using the Algorithm for Liver Function Investigations (ALFI)
Source: PLoS One. 2012 Dec 14;7(12):e50965. doi: 10.1371/journal.pone.0050965 (PMC3522690; doi:10.1371/journal.pone.0050965)
Supplement: Appendix S3 — Calibration of the final model. (DOC) [file pone.0050965.s003.doc]

Appendix S3

Calibration of the final model

The calibration curve (Figure S1) shows reasonable agreement between the predicted and actual probabilities. There is evidence of some over-prediction between predicted probabilities 0.6 to 0.8 but overall the plot indicates that the model predicts satisfactorily across the range of probabilities. The calibration slope did not deviate significantly from 1 (slope = 1.023 (95% CI 0.995 to 1.051); p=0.11) suggesting no evidence of over-fitting.

**Figure S1 Calibration plot showing the relationship between the predicted and actual (Kaplan-Meier) probabilities of survival.**


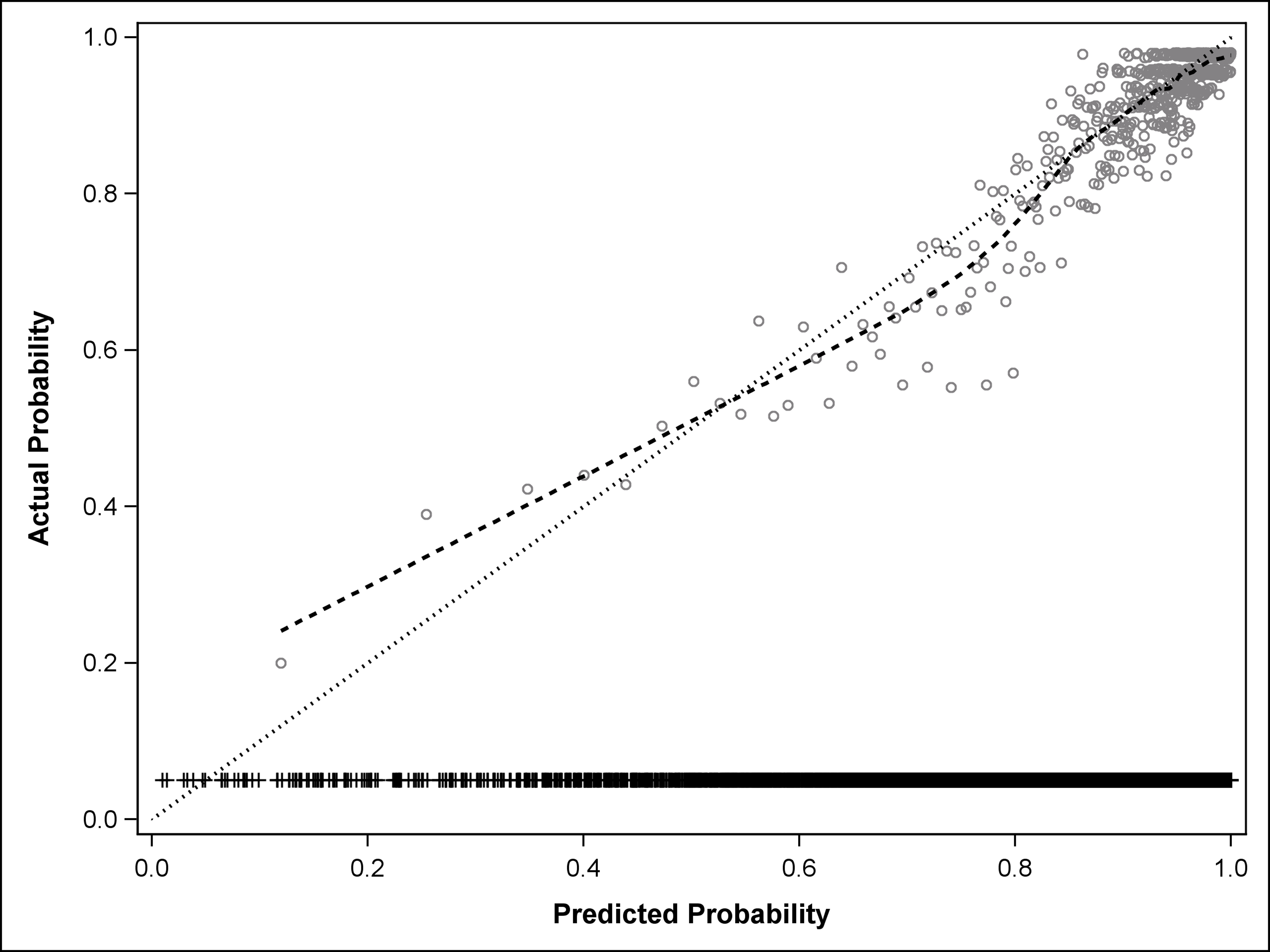


Note: Dashed line represents the smooth non-parametric Loess calibration curve; the circles represent the mean probabilities for subgroups of patients; the dotted line represents the perfect relationship; the plus symbols represent the spread of patients across predicted probabilities.
