# Supplementary material for: The Utility of Liver Function Tests for Mortality Prediction within One Year in Primary Care Using the Algorithm for Liver Function Investigations (ALFI)
Source: PLoS One. 2012 Dec 14;7(12):e50965. doi: 10.1371/journal.pone.0050965 (PMC3522690; doi:10.1371/journal.pone.0050965)
Supplement: Appendix S4 — Discrimination and calibration of the model applied to the external cohort. (DOC) [file pone.0050965.s004.doc]

Appendix S4

Discrimination and calibration of the model applied to the external cohort

The C-statistic for the final model applied to the external cohort was 0.86 (95% CI 0.79 to 0.90). When a model was fitted to the external cohort using the same covariates (but not the same parameter estimates) as the final model, the C-statistic was 0.86 (95% CI 0.80 to 0.91) meaning that the discrimination was no better for a model with the external cohort’s own parameter estimates than for the final model with its own parameter estimates applied to the external cohort. The z-test only showed a significant difference between the three regression coefficients for history of respiratory disease, albumin, and bilirubin (see Table S4).

The calibration plot (Figure S2a) for the final model applied to the external cohort showed slight evidence of over-fitting, and the calibration slope showed borderline deviation from 1 at the 5% level, (slope = 0.929 (95% CI 0.856 to 1.001); p=0.055). The model was recalibrated for the external cohort by adding the intercept and subtracting the deviation of the slope of Xβ i.e. (0.948 – 0.071*Xβ). The recalibrated plot showed a marked improvement (Figure S2b).

Table S4 Parameter estimates (95% CI) for the external validation cohort and their comparison with the final model parameter estimates using the z-test.

| **Covariate** | **Parameter Estimate**  **(95% CI)** | **z-test**  **p-value** |
| --- | --- | --- |
| Intercept | 18.851 (12.294 to 25.409) § | 0.31 |
| Albumin | 0.154 (0.126 to 0.181) § | **0.01** |
| Cancer (yes *v* no) | -3.759 (-5.280 to -2.238) § | 0.38 |
| Age at baseline X Cancer | 0.041 (0.021 to 0.061) § | 0.47 |
| Log (ALP) | -2.650 (-4.023 to -1.277) § | 0.54 |
| Age at baseline | -0.179 (-0.265 to -0.093) § | 0.27 |
| Respiratory disease (yes *v* no) | 0.025 (-0.359 to 0.409) | **0.004** |
| Stroke (yes *v* no) | -0.445 (-0.756 to -0.134) ‡ | 0.46 |
| Bilirubin (mildly raised *v* normal) | 0.138 (-0.244 to 0.520) | **0.01** |
| Log (transaminase) | 0.603 (-0.577 to 1.782) | 0.28 |
| Log (GGT) | -0.298 (-0.501 to -0.095) ‡ | 0.29 |
| Age at baseline X Log (ALP) | 0.025 (0.007 to 0.043) ‡ | 0.42 |
| Gender (Male *v* Female) | -1.770 (-3.110 to -0.431) ‡ | 0.43 |
| Statins (yes *v* no) | 0.238 (-0.011 to 0.487) ^+^ | 0.06 |
| Deprived (yes *v* no) | -0.002 (-1.539 to 1.536) | 0.23 |
| Renal disease (yes *v* no) | -0.602 (-1.083 to -0.121) † | 0.32 |
| IHD (yes *v* no) | -0.212 (-0.459 to 0.035) ^+^ | 0.75 |
| Age at baseline X Deprived | -0.003 (-0.023 to 0.017) | 0.19 |
| Age at baseline X Log (transaminase) | 0.002 (-0.014 to 0.017) | 0.19 |
| Gender X Age at baseline | 0.016 (-0.001 to 0.034) ^+^ | 0.54 |
| Scale | 1.292 (1.007 to 1.577) § | **0.004** |
| Shape | 0.582 (0.336 to 0.829) § | 0.19 |

^+^ 0.05<p≤0.1; † 0.01<p≤0.05; ‡ 0.001<p≤0.01; § p≤0.001

IHD ischaemic heart disease; GGT = gamma-glutamyl transferase; ALP = alkaline phosphatase

**Figure S2 Calibration plot showing the relationship between the predicted and actual probabilities of survival for a) the final model applied to the external validation cohort; b) the recalibrated final model applied to the external validation cohort.**


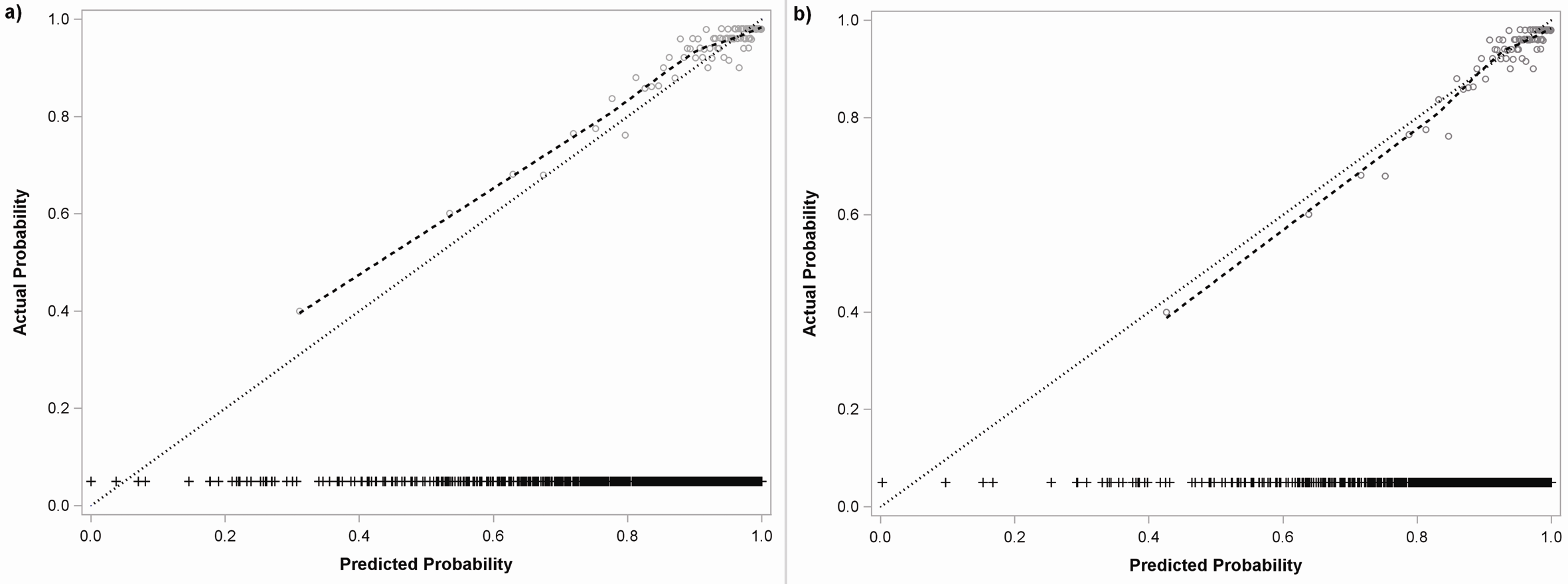


Note: Dashed line represents the smooth non-parametric Loess calibration curve; the circles represent the mean probabilities for subgroups of patients; the dotted line represents the perfect relationship; the plus symbols represent the spread of patients across predicted probabilities.
