# Supplementary material for: The Utility of Liver Function Tests for Mortality Prediction within One Year in Primary Care Using the Algorithm for Liver Function Investigations (ALFI)
Source: PLoS One. 2012 Dec 14;7(12):e50965. doi: 10.1371/journal.pone.0050965 (PMC3522690; doi:10.1371/journal.pone.0050965)
Supplement: Appendix S5 — How to calculate the predicted probability of mortality within T days (where 1≤T≤365). (DOC) [file pone.0050965.s005.doc]

**Appendix S5**

How to calculate the predicted probability of mortality within T days (where 1≤T≤365)

| **Predictor** | **Description** | **Possible values** |
| --- | --- | --- |
| Gender | Male or female | =1 if Male  =0 if Female |
| Age | Age | 16 to 103 |
| Deprived | Deprivation based on Carstairs category linked to postcode | =1 if Yes (i.e. Carstairs categories 4 to 7)  =0 if No (i.e. Carstairs categories 1 to 3) |
| IHD | Patient has a history of ischaemic heart disease (IHD) | =1 if Yes  =0 if No |
| Renal | Patient has a history of renal disease | =1 if Yes  =0 if No |
| Respiratory | Patient has a history of respiratory disease | =1 if Yes  =0 if No |
| Stroke | Patient has a history of stroke | =1 if Yes  =0 if No |
| Cancer | Patient has a history of cancer | =1 if Yes  =0 if No |
| Statins | Prescribed statins in the previous 3 months | =1 if Yes  =0 if No |
| ALP | Alkaline phosphatase result | 9U/L to ∞ |
| GGT | Gamma-glutamyltransferase result | 2U/L to ∞ |
| Albumin | Albumin result | 14 to 63g/L |
| Transaminase | Alanine transaminase/aspartate aminotransferase result | 4U/L to ∞ |
| Bilirubin | Bilirubin result categorised as normal or mildly raised. Exclude anyone with bilirubin >35μmol/L. | =0 if 0-15μmol/L (female); 0-17μmol/L (male)  =1 if 16-35μmol/L (female); 18-35μmol/L (male) |

First calculate the linear predictor, ***Xβ***, using the possible values contained within the table above:

***Xβ***=15.1579 – ***A*** + ***B***

where:

***A***=1.1838*Gender + 0.1268*Age + 0.9986*Deprived + 0.2592*IHD + 0.9693*Renal + 0.6054*Respiratory + 0.5873*Stroke + 4.5382*Cancer + 2.1793*LOG_e_(ALP) + 0.4526*LOG_e_(GGT+0.5) + 0.4031*Bilirubin + 0.0100*Age*LOG_e_(Transaminase)

and

***B***= 0.6166*Statins + 1.3229*LOG_e_(Transaminase) + 0.1937*Albumin + 0.0103*Age*Gender + 0.0111*Age*Deprived + 0.0500*Age*Cancer + 0.0172*Age*LOG_e_(ALP)

$$\text{U}=\left(\text{T}*\exp\left(-\text{Xβ}\right)\right)^{0.5674}$$

where ***T***=follow-up time, choose from 1 to 365 days

**Predicted probability of mortality within *T* days** = $GAMMADIST\left(6.0115*U^{0.4079},6.0115,1,TRUE\right)$

i.e. the probability from the generalised gamma distribution. This can be calculated using the *GAMMADIST* function in Microsoft Excel.

**A worked example**

Suppose we want to calculate the probability of mortality within 1 year for a 55 year-old male living in an affluent area. He has no history of cancer, IHD, renal disease, respiratory disease and stroke, and has the following LFT results: ALP=137U/L, albumin=28g/L, bilirubin=9μmol/L, GGT=86U/L and Transaminase=41U/L.

***Xβ***=15.1579 – ***A*** + ***B***

where, inserting the patient’s information into the above formula:

***A***=1.1838*1 + 0.1268*55 + 0.9986*0 + 0.2592*0 + 0.9693*0 + 0.6054*0 + 0.5873*0 + 4.5382*0 + 2.1793*LOG_e_(137) + 0.4526*LOG_e_(86.5) + 0.4031*0 + 0.0100*55*LOG_e_(41)

and

***B***= 0.6166*0 + 1.3229*LOG_e_(41) + 0.1937*28 + 0.0103*55*1 + 0.0111*55*0 + 0.0500*55*0 + 0.0172*55*LOG_e_(137)

***Xβ***=15.1579 – 22.9410 + 15.5571

= 7.774

Therefore,

$$U=\left(365*\exp\left(-7.774\right)\right)^{0.5674}$$

= 0.3453

**Predicted probability of mortality within 365 days** = $GAMMADIST\left(6.0115*U^{0.4079},6.0115,1,TRUE\right)$ =$GAMMADIST\left(6.0115*0.3453^{0.4079},6.0115,1,TRUE\right)$

= $GAMMADIST\left(3.8959,6.0115,1,TRUE\right)$

= 0.1974

using the *GAMMADIST* function in Microsoft Excel.
